# Mechanism and kinetics of chlorpyrifos co-metabolism by using environment restoring microbes isolated from rhizosphere of horticultural crops under subtropics

**DOI:** 10.3389/fmicb.2022.891870

**Published:** 2022-07-26

**Authors:** Govind Kumar, Shatrohan Lal, Sumit K. Soni, Shailendra K. Maurya, Pradeep K. Shukla, Parul Chaudhary, A. K. Bhattacherjee, Neelima Garg

**Affiliations:** ^1^Indian Council of Agricultural Research (ICAR)–Central Institute for Subtropical Horticulture, Lucknow, Uttar Pradesh, India; ^2^Department of Animal Biotechnology, Indian Council of Agricultural Research (ICAR)–National Dairy Research Institute, Karnal, Haryana, India

**Keywords:** chlorpyrifos, biodegradation, plant growth promotion, consortium, ERM C-1

## Abstract

The indiscriminate use of organophosphate insecticide chlorpyrifos in agricultural crops causes significant soil and water pollution and poses a serious threat to the global community. In this study, a microbial consortium ERM C-1 containing bacterial strains *Pseudomonas putida* T7, *Pseudomonas aeruginosa* M2, *Klebsiella pneumoniae* M6, and a fungal strain *Aspergillus terreus* TF1 was developed for the effective degradation of chlorpyrifos. Results revealed that microbial strains were not only utilizing chlorpyrifos (500 mg L^–1^) but also coupled with plant growth-promoting characteristics and laccase production. PGP traits, that is, IAA (35.53, 45.53, 25.19, and 25.53 μg mL^–1^), HCN (19.85, 17.85, 12.18, and 9.85 μg mL^–1^), and ammonium (14.73, 16.73, 8.05, and 10.87 μg mL^–1^) production, and potassium (49.53, 66.72, 46.14, and 52.72 μg mL^–1^), phosphate (52.37, 63.89, 33.33, and 71.89 μg mL^–1^), and zinc (29.75, 49.75, 49.12, and 57.75 μg mL^–1^) solubilization tests were positive for microbial strains T7, M2, M6, and TF1, respectively. The laccase activity by ERM C-1 was estimated as 37.53, 57.16, and 87.57 enzyme U mL^–1^ after 5, 10, and 15 days of incubation, respectively. Chlorpyrifos degradation was associated with ERM C-1 and laccase activity, and the degree of enzyme activity was higher in the consortium than in individual strains. The biodegradation study with developed consortium ERM C-1 showed a decreased chlorpyrifos concentration from the 7th day of incubation (65.77% degradation) followed by complete disappearance (100% degradation) after the 30th day of incubation in the MS medium. First-order degradation kinetics with a linear model revealed a high *k ^–day^* value and low *t*_1/2_ value in ERM C-1. The results of HPLC and GC-MS analysis proved that consortium ERM C-1 was capable of completely removing chlorpyrifos by co-metabolism mechanism.

## Introduction

Chlorpyrifos, an acetylcholinesterase inhibitor, causes neurological diseases in sensitive groups, particularly youngsters and the elderly population (Farkhondeh et al., 2020; Huang et al., 2021; Rahman et al., 2021). Chlorpyrifos pollution in the environment, including soil, air, and water, has been extensively investigated due to increased usage and greater diffusion impacting even unanticipated sites (Bhatt et al., 2021; Farhan et al., 2021; Huang et al., 2021; Kumar et al., 2021; Rahman et al., 2021). The half-life of chlorpyrifos in the soil is generally 60–120 days, while it can range from 15 to over 365 days depending on the soil type and other environmental factors (Kumar et al., 2021). In comparison to other approaches, such as physical and chemical remediation, biodegradation of chlorpyrifos in the soil is usually regarded as a low-cost, environmentally friendly, and less energy-demanding biotechnological strategy with a high benefit-to-cost ratio for cleaning up the polluted soil ecosystems (Bhatt et al., 2021; Elshikh et al., 2021; Kumar et al., 2021).

Microorganisms having the ability to degrade chlorpyrifos have been reported mostly from polluted environments. The most common pesticide-degrading microbes described in the literature are *Bacillus*, *Burkholderia*, *Rhodococcus*, *K. pneumoniae*, and *Pseudomonas* sp.; however, *Pseudomonas* sp. has the most reports for the degradation of aromatic compounds (Bhatt et al., 2021; Farhan et al., 2021; Huang et al., 2021; Kumar et al., 2021; Rahman et al., 2021). Recently, Chishti et al. (2021) tested chlorpyrifos degradation in different soil-slurry systems using *Enterobacter* sp. SWLC2 strains. Similarly, Uniyal et al. (2021) carried out a microcosm study of chlorpyrifos biodegradation using rhizobacterial consortium ECO-M on apple. Lin et al. (2022) have developed a bacterial consortium, ZQ01, for the effective degradation of acephate and their toxic intermediate product methamidophos.

Elshikh et al. (2021) and Kumar et al. (2021c) have reported that *Bacillus cereus* and *Klebsiella pneumoniae* were the most prominent biodegraders of chlorpyrifos in submerged fermentation and in the soil, respectively. Apart from bacteria, fungal strains have also been reported for chlorpyrifos degradation individually and in combination. A recent report has suggested the application of fungal strains *Byssochlamys spectabilis* C1 and *Aspergillus fumigatus* C3 for chlorpyrifos degradation in liquid CDM and in the soil (Kumar et al., 2021). Furthermore, chlorpyrifos metabolism by microorganisms is known to be a three-step process in which chlorpyrifos is first converted into a bioavailable form by reducing the surface tension of the pesticide with the help of biosurfactants produced by bacterial strains (Kumar et al., 2015). These surface tension-reducing microorganisms have the property to enhance the bioavailability of hydrophobic pesticides for microbial activity. As pesticides become bioavailable, microbes further secrete other catabolic enzymes and couple with sugar or amino acid molecules (dechlorination), and finally, get converted into non-toxic compounds like carbon dioxide and water. Several catabolic enzymes like dioxygenases, hydrolases/esterases, glutathione S-transferases (GSTs), and cytochrome P450 take part in the complete metabolism of chlorpyrifos. Additionally, the mixed-function of GSTs with oxidases is involved in the second metabolic phase (Foong et al., 2020; Ambreen and Yasmin, 2021; Aswathi et al., 2021; Nandhini et al., 2021; Soares et al., 2021; Uniyal et al., 2021). Phosphotriesterases (PTE), organophosphate hydrolases (OPH), methyl parathion hydrolase (MPH), and chlorpyrifos hydrolase (CPH) are the most often described enzymes for chlorpyrifos biodegradation (Zhao et al., 2021; Bhende et al., 2022).

Numerous investigations on chlorpyrifos degradation using pure microbial cultures have been carried out in recent years (Gaonkar et al., 2019; Farhan et al., 2021). However, only a few microbial strains have been identified that can effectively degrade chlorpyrifos in both aqueous and soil environments (Ambreen and Yasmin, 2021; Kumar et al., 2021; Uniyal et al., 2021). Furthermore, the application of single culture or artificial consortia as a remediating agent has not been found to be the most appropriate approach in the bioremediation process occurring in the natural environment, as it occurs in real nature and depends on the collaborative metabolic functions of diversified indigenous microbial communities (Kumar et al., 2021b).

An efficient degradation system can be established by developing a consortium of distinct domain microorganisms isolated from a real polluted environment with the competence to utilize the chemical of interest as the carbon source due to their ability for synergistic metabolism. With the understanding of knowledge aforesaid, the effort has been made to develop the microbial consortia among two different domain microbes for the effective remediation of chlorpyrifos from subtropical agriculture land, which is not possible by individual single microorganisms.

## Materials and methods

### Chemicals

Analytical grade Chlorpyrifos (purity 99.9%) was purchased from Sigma-Aldrich Co. (St. Louis, MO, United States). Other chemicals used in this study were also analytical grade quality and purchased from Merck, India. Media used were purchased from Hi-Media Laboratories, Mumbai, India. Mineral salt medium (stocktickerMSM) used in this study contained (g L^–1^): (NH_4_)_2_SO_4_ 2.0, MgSO_4_.7H_2_O 0.2, CaCl_2_.2H_2_O 0.01, FeSO_4_.7H_2_O 0.001, Na_2_HPO_4_.12H_2_O 1.5, and KH_2_PO_4_ 2.0. The stock solution of chlorpyrifos (2150 g L^–1^) and 3, 5, 6-trichloro-2-pyridinol (100 mg L^–1^) was prepared in a mixture of acetone + n-hexane (50:50) and passed through 0.22-μm syringe filters.

### Sampling, isolation, and characterization of chlorpyrifos-degrading microorganisms

For sampling, a survey was conducted to determine the pesticide application in subtropical regions. Two locations, ICAR-CISH farm (26° 45′ - 27° 10′ N, 80° 30′–80° 55′ E) and progressive farmers field of Malihabad region (26° 91′–27° 21′ N, 80° 70′–80° 95′ E), were selected on the basis of frequent pesticide applications. Nine rhizosphere soil samples were collected from three different crops, namely, *Solanum lycopersicum*, *Solanum melongena*, and *Capsicum annum* (bell pepper) plant. Twenty grams of soil sample was transferred into a 250 mL Erlenmeyer flask containing 50 mL of MS medium amended with 100 mg L^–1^ of chlorpyrifos. The flasks were incubated at 30 ± 2°C with shaking at 110 rpm. After 5 days of incubation, 5 mL (pioneer acclimatized) of culture aliquot was transferred to a freshly prepared MS medium containing (100 mg L^–1^) chlorpyrifos. The flasks were again incubated for 5 days under the previously mentioned conditions. By following the above-mentioned method, enriched culture was shifted periodically into the liquid MS medium with increasing concentrations of chlorpyrifos up to 200, 300, 400, and 500 mg L^–1^ over 5 following days at 30 ± 2°C temperature and 100 rpm shaking. Five successive transfers were performed to screen for potent chlorpyrifos-degrading strains. From the last flask, 1 mL of culture was serially diluted, and 100 μL was spread on solid Luria-Bertani (LB) agar and potato dextrose agar plate amended with 500 mg L^–1^ chlorpyrifos for bacterial and fungal growth, respectively. Furthermore, plates were incubated at 30 ± 2°C for bacterial growth and 28 ± 2°C for fungal growth. The grown colonies with uniform morphologies were purified on solid agar plates. Fungal and bacterial colonies with a high proclivity for tolerating chlorpyrifos toxicity were chosen for further experiments. Four microorganisms (three bacterial strains T7, M2, and M6, and one fungal strain TF1), capable of growing at maximum chlorpyrifos concentration (500 mg L^–1^), were selected for further characterization. Selected microbes were identified through ribotyping by amplifying the 16S rRNA gene for bacterial strains using forward 27F (5′-AGAGTTTGATCCTGGCTCAG-3′) and 1392R (5′-GGTTACCTTGTTACGACTT-3′) primers, while for fungal strain, ITS regions were amplified using primers ITS1 (5′-TCCGTAGGTGAACCTGCGG-3′) and ITS4 (5′-GCTGCGTTCTTCATCGATGC-3′), respectively. The amplified genes were sequenced using Sanger dideoxy sequencing method. The obtained sequences were submitted to the NCBI Genbank database^1^ under the accession numbers MW172266, MW228078, MW228061, and MZ268151, respectively. The phylogenetic analysis was performed using MEGA version 5.2 software. After the BLAST analysis, the FASTA form of a sequence of most similar organisms along with nearest-neighbor sequences from the NCBI database was downloaded. Apart from this, one analog sequence of other genera was also taken for the out-group purpose. The downloaded sequences were aligned by the inbuilt ClustalW alignment tool of MEGA version 5.2 software.

The evolutionary history was inferred by using the maximum likelihood method and the General Time-Reversible Model with bootstrap replications of 1,000 (Nei and Kumar, 2000). The tree with the highest log-likelihood value is shown in Supplementary Figures 1–3. The percentage of trees in which the associated taxa clustered together is shown next to the branches. Initial tree(s) for the heuristic search was obtained automatically by applying Neighbor-Join and BioNJ algorithms to a matrix of pairwise distances estimated using the maximum composite likelihood (MCL) approach, and then selecting the topology with a superior log-likelihood value. The tree is drawn to scale, with branch lengths measured in the number of substitutions per site.

### Plant growth-promoting attributes of chlorpyrifos-degrading microorganisms

The selected microbes were tested for plant growth-promoting attributes qualitatively and quantitatively following the standard methods described by previous researchers. Phosphate solubilization was assessed by the method of Nautiyal (1999), while Zn solubilizing activity was tested following the method of Mumtaz et al. (2017). K solubilization was assessed by the method described by Bagyalakshmi et al. (2017), HCN production by Lorck (1948), and ammonia production by Cappuccino and Sherman (2005). Moreover, IAA production was checked by following the method described by Bric et al. (1991) on their respective media. All the tests were performed in triplicates.

### Laccases production by chlorpyrifos-degrading microorganisms

In order to test the activity of laccases, Luria-Bertani agar plates supplemented with guaiacol (0.01% w/v) as a substrate for laccase and 0.35 mM CuSO_4_ were inoculated with the selected isolates. Plates were incubated at 30 ± 2°C for 24 h, and the development of yellow to brown colored zones around the colonies indicates a positive test. Furthermore, quantitative estimation of the activity of laccases was done as per the method described by Abd El Monssef et al. (2016) which was later modified by Kumar et al. (2021). Briefly, fungal and bacterial strains were grown separately in their respective liquid media, and the supernatant was reacted with sodium acetate buffer (10 mM, pH 4.5), guaiacol (1 mM), and CuSO_4_ (2 mM). Blank with water served as control. The optical density in terms of absorbance of the reaction mixture was measured at 410 nm with the help of a spectrophotometer (Evolution 201, Thermo Fisher Scientific, Waltham, MA, United States). The activity of laccases was estimated (U mL^–1^) using the following equation:


(1) $$E.A=A\times \frac{V}{t\times \varepsilon \times v}$$

where E.A is the enzyme activity in units per mL, A is the absorbance at 410 nm, *V* is the total mixture volume (mL), *v* is the enzyme volume (mL), t is the incubation time (min), and ε is the extinction coefficient of guaiacol (0.674 μm cm^–1^).

### Screening of biosurfactant production by chlorpyrifos-degrading microbes

Chlorpyrifos-degrading microorganisms (T7, M2, M6, and TF1) were evaluated for biosurfactant production using liquid MSM fed with glucose 2% (w/v) and yeast extract 0.25% (w/v). It was assumed that biosurfactants contribute to the increase of the aqueous phase partitioning of chlorpyrifos, resulting in increased chlorpyrifos bioavailability for microbial activity.

#### Emulsification activity (E_24_)

The E_24_ activity was checked by homogenizing 2 mL of chlorpyrifos (21,500 g L^–1^) and 2 mL of cell-free supernatant followed by high-speed vortexing for 2 min. The emulsification activity after 24 h was determined using the following formula:


$${\text{E}}_{24}\left(\%\right)=\frac{\mathrm{Total}\,\mathrm{height}\,\mathrm{of}\,\mathrm{the}\,\mathrm{emulsified}\,\mathrm{layer}\,\left(\text{cm}\right)}{\mathrm{Total}\,\mathrm{height}\,\mathrm{of}\,\mathrm{the}\,\mathrm{liquid}\,\mathrm{layer}\,\left(\text{cm}\right)}\times 100$$

#### Foaming test

The foaming test was performed by following the method of Abouseoud et al. (2008), which was later modified by Ratna and Kumar (2022). The production of foam by 96-h old culture, when shaken gently for 2 min, indicated positive results for foam production. The stability of the foam was monitored by observing it for 2 h.

#### Drop-collapse test

The test was performed by following the qualitative drop-collapse test described by Bodour and Miller-Maier (1998). Chlorpyrifos concentration of 21,500 gL^–1^ was used for this study. About 2 μL of chlorpyrifos was inoculated into 96-well microplates and allowed to equilibrate for 24 h. After 48 h, 2 μL of culture supernatant was transferred to the chlorpyrifos-coated well sections, and the drop size was measured using a magnifying glass after 1 min. A flat drop was considered as a positive result for biosurfactant production, while rounded drops were regarded as negative results, indicating a lack of biosurfactant production.

### Development of microbial consortium for chlorpyrifos degradation

The consortium was developed based on the compatibility test between the microbes. Two methods, dual culture plate and crowded plate assay, were followed for the compatibility test (Muniaraj et al., 2008; Xu and Kim, 2014). Microbes showing compatible growth were used to develop the microbial consortium. Three bacterial strains (*Pseudomonas putida* T7, *Pseudomonas aeruginosa* M2, and *Klebsiella pneumoniae* M6) and one fungal strain (*Aspergillus terreus* TF1) were combined to develop a consortium and named as environment restoring microbes (ERM C-1). For this, bacterial and fungal strains were individually inoculated in a liquid nutrient broth medium, and flasks were incubated at 30 ± 2°C for 72 h. Furthermore, cell biomass was collected after centrifugation at 12,879 g for 5 min. The cell pellets were washed two times with sterile distilled water and finally suspended in sterile saline solution (0.9% NaCl). The cell density (absorbance) was maintained at 1.0 OD (600 nm) with the help of a spectrophotometer (Evolution 201, Thermo Fisher Scientific United States). It was assumed that both domain microbes (bacteria and fungi) were maintained in equal quantity while preparing the consortium (Uniyal et al., 2021).

### Biodegradation study of chlorpyrifos in liquid MS medium, and natural and sterile soil system

For chlorpyrifos biodegradation, a lab-scale batch experiment was performed in 250 mL Erlenmeyer flasks containing 100 mL MSM amended with 500 mg L^–1^ chlorpyrifos. Selected strains were inoculated individually and with the developed consortium. Flasks without inoculated microbes served as control. Furthermore, flasks were wrapped with brown paper to evade photolysis and were incubated in a rotatory shaker at 30 ± 2°C and 120 rpm speed. About 20 mL of culture aliquots were withdrawn from every flask at 7-, 15-, and 30-day intervals, and the supernatant was used for the extraction of residual chlorpyrifos and further measured using HPLC (Nexer-R, SIL-30ACMP, Shimadzu Japan) and GC-MS (TQ 8050 Nexis Shimadzu Japan) techniques. The bacterial growth was measured at 600 nm, while the fungal growth was measured at 310 nm with the help of a spectrophotometer (Abdul Manan and Webb, 2018).

For biodegradation of chlorpyrifos in sterile and natural soil system, 250 g of agriculture soil was filled in sterile plastic-wrapped paper pots. Chlorpyrifos (500 mg kg^–1^) was amended to the soil (sterile and natural) and mixed thoroughly to ensure a uniform concentration of chlorpyrifos. Individual strains and developed consortium (Section Screening of biosurfactant production of chlorpyrifos degrading microbes) were inoculated at a concentration of 1 × 10^8^cfu g^–1^ into chlorpyrifos-spiked soils. The sterile soil pots were wrapped with 100 mm of clinging plastic wrap to avoid environmental contamination. The samples (20 g) were recovered in triplicates at 7-, 15-, and 30-day intervals and extracted with acetonitrile. The chlorpyrifos residue was analyzed with the help of HPLC and GC-MS (Bhatt et al., 2021).

### Kinetics of chlorpyrifos biodegradation

In order to estimate biodegradation kinetics in different systems, first-order degradation kinetics was applied to the experimental data. The first-order degradation equation is mentioned below:


(2) $$C\,t=C_{0}\,e^{-k\,t}$$

where Log “C” (chlorpyrifos residue in the particular medium) is calculated against time “t” to determine the “k” value, while “C_0_” denotes the initial concentration of chlorpyrifos in different systems (MSM, SS, and NS). “Ct” represents chlorpyrifos concentration at reaction time “t,” and “k” represents the constant rate of chlorpyrifos degradation day^–1^. For the estimation of the half-life of chlorpyrifos biodegradation in different systems, a graph was plotted between time “t” and constant “k.” The equation for the half-life is as below:


(3) $$t_{1/2}=\frac{L\,n\,2}{k}$$

### Soil enzyme dynamics of chlorpyrifos-treated soils

Two soil enzymes, dehydrogenase (DHA) and fluorescein diacetate (FDA), directly related to soil microbial activities were assessed in chlorpyrifos-treated soils. The DHA activity was tested by following the standard protocol reported by Casida et al. (1964), while FDA activity was checked by the method developed by Schnrer and Rosswall (1982), later modified by Adam and Duncan (2001). Dehydrogenase activity is normally found in soil as part of the oxidative reactions that occur within live cells, and hence its measurement represents only intracellular enzyme activity, making it a good indication of microbial activity. However, the hydrolysis activity of fluorescein diacetate is a non-specific assay in which lipase, esterase, and protease classes of enzymes hydrolyze fluorescein by cleaving the ring, so it was assumed that they may also take part in the cleavage of bonds present in chlorpyrifos ring.

### Statistical analysis

For the kinetic study of chlorpyrifos biodegradation, a statistical analysis software package (Origin Pro 2018b, MA, United States) was used. Three replicates of each sample were used in the statistical analysis of the biodegradation data. The data were also validated by one-way analysis of variance (ANOVA) and Duncan’s multiple range test (DMRT) to compare the mean values. The IBM-SPSS program (version 25, IBM, New York, NY, United States) was used to perform a *post-hoc* DMRT test analysis. Statistical significance was calculated using the lowest significance differences (LSDs) at *P* ≤ *0.05* to analyze the differences among treatments.

## Results

### Isolation and characterization of chlorpyrifos-degrading microbes

In this study, a sum of 21 different bacterial and four fungal isolates was screened from nine different samples at the primary stage in the presence of 100 mg L^–1^ of chlorpyrifos amended medium. Further screening was done on the basis of the growth of microbes at a maximum concentration (500 mg L^–1^) of chlorpyrifos and plant growth-promoting traits. Results revealed that out of 21 bacterial and four fungal isolates, three bacterial strains (T7, M2, and M6) and one fungal strain (TF1) were capable of growing at the maximum concentration (500 mg L^–1^) of chlorpyrifos and showed multiple plant growth-promoting characteristics. Apart from this, all these isolates (T7, M2, M6, and TF1) were also tested for growth in the presence of 250 ppm of TCP. Observed results showed a luxuriant growth of all the four isolates in the presence of TCP amended medium. Finally, identification of the strains, including both bacteria and fungi, was carried out using 16S rRNA and ITS molecular approaches, respectively. The molecular identification through homology searching and BLAST analysis (Table 1) revealed that the isolated bacterial strains, that is, M2 and T7, belonged to *Pseudomonas*, while strain M6 belonged to *Klebsiella* genera and fungal strain TF1 belonged to *Aspergillus* genera. The phylogenetic position of these strains with other related organisms has been depicted in Supplementary files (1, 2 and 3).

**TABLE 1:** Homology search of isolated Plant growth promoting rhizobacteria (PGPRs).

Isolated PGPRs strains	Identification	GenBank accession no.	Similar organism	Accession number	Sequence similarity (%)
T7	*Pseudomonas putida*	MW172266	*Pseudomonas putida*	DQ112329	97%
M2	*Pseudomonas aeruginosa*	MW228078	*Pseudomonas aeruginosa*	OK668300	97%
M6	*Klebsiella pneumoniae*	MW228061	*Klebsiella pneumoniae*	MT102629	97%
TF-1	*Aspergillus terreus*	MZ 268151	*Aspergillus terreus*	KM401402	96.84%

### Plant growth-promoting characteristics of selected strains

**FIGURE 1:**
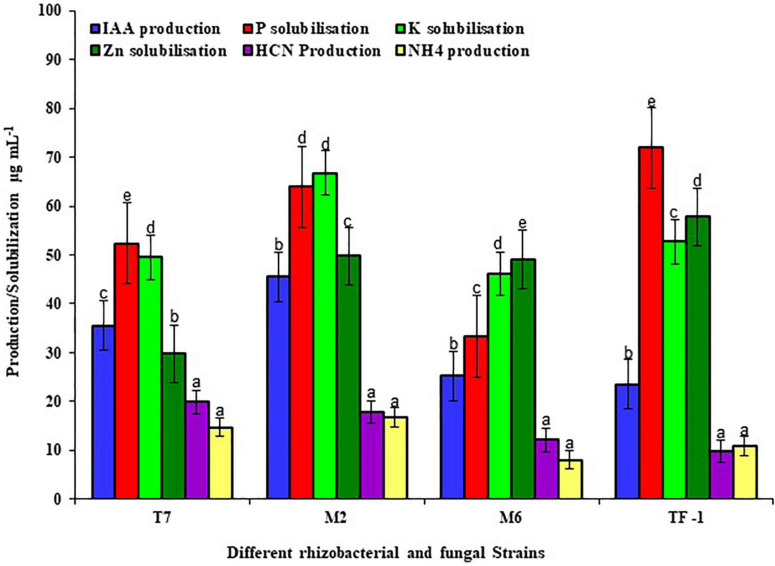
Different plant growth-promoting properties of rhizobacterial and fungal strains. The results were the average of five replicates (*n* = 5). Bars represent standard error. Significant differences based on the analysis variance (ANOVA) are shown by different letters above the error bars, followed by the *post-hoc* DMRT test (*p* ≤ 0.05) using the software SPSS.

To test the plant growth-promoting characteristics, the selected strains were grown on a specific medium to detect the positive characteristics. Results revealed that strains T7, M2, M6, and TF1 showed positive results for phosphate solubilization, potassium solubilization, zinc solubilization, IAA production, HCN production, and ammonia production. Furthermore, quantitative estimation revealed that T7 solubilized 52.37 ± 2, 49.53 ± 2, and 29.75 ± 2 μg mL^–1^ of phosphate, potassium, and zinc, respectively, while producing 35.53 ± 2, 19.85 ± 2, and 14.73 ± 2 μg mL^–1^ of IAA, HCN, and ammonium, respectively. Similarly, M2 solubilized 63.89 ± 2, 66.72 ± 2, and 49.75 ± 2 μg mL^–1^ of phosphate, potassium, and zinc, respectively, and produced 45.33 ± 2, 17.30 ± 2, and 16.37 ± 2 μg mL^–1^ of IAA, HCN, and ammonium, respectively. Strain M6 solubilized 33.33 μg mL^–1^ of phosphate, 46.14 ± 2 μg mL^–1^ of potassium, and 49.12 ± 2 μg mL^–1^ of zinc in an aqueous medium, while 25.19 ± 2 μg mL^–1^ of IAA, 12.18 ± 2 μg mL^–1^ of HCN, and 8.05 ± 2 μg mL^–1^ of ammonium were produced. Moreover, fungal strain TF1 showed positive PGP characteristics and solubilized 71.89 ± 2, 52.72 ± 2, and 57.75 ± 2 μg mL^–1^ of phosphate, potassium, and zinc, respectively, and 23.53 ± 2, 9.85 ± 2, and 10.87 ± 2 μg mL^–1^ of IAA, HCN, and ammonium were produced, respectively (Figure 1).

### Estimation of laccase production by selected microorganisms

**FIGURE 2:**
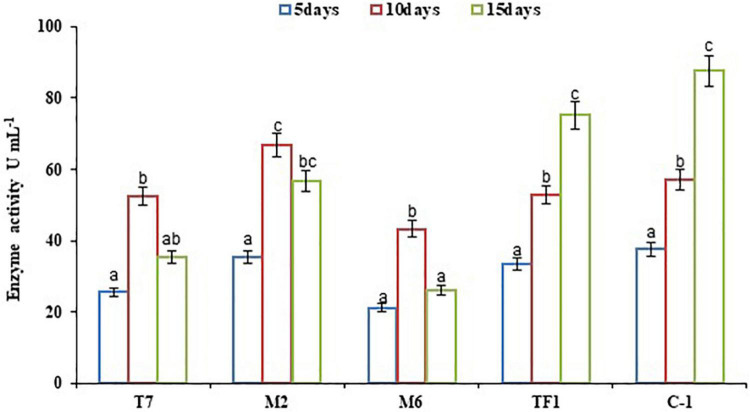
Laccase activity by individual strains and developed consortium. The results were the average of five replicates (*n* = 5). Bars represent the standard error. Significant differences based on the analysis variance (ANOVA) are shown by different letters above the error bars, followed by the *post-hoc* DMRT test (*p* ≤ 0.05) using the software SPSS.

Microbial enzymes are considered a safe, eco-friendly, and green method for the breakdown of complex substances in water and terrestrial ecosystem. The microbial enzyme tests are employed in the soil to investigate the pattern of degradation of complicated compounds like chemical pesticides. The results revealed that bacterial strains (T7, M2, and M6) showed maximum laccase activity within 10 days of incubation, and later a decrease in enzyme production had been observed. Strains T7, M2, and M6 produced 25.53–52.37, 35.53–66.89, and 21.19–43.33 U mL^–1^ enzyme, respectively, between 5 and 10 days of incubation. However, fungal strain TF1 reached the maximum enzyme production (75.12 U mL^–1^) after the 15th day of incubation, and thereafter the rate of enzyme production decreased, which might be due to the lack of substrate in the medium or due to the feedback inhibition. Furthermore, enzyme production was greater (87.57 U mL^–1^) in the consortium ERM C-1 group than in groups treated with individual strains (Figure 2).

### Biosurfactant production of chlorpyrifos-degrading microorganisms

The results of this study revealed that strains T7 and M2 were positive for emulsification activity (E24), foaming test, and drop-collapse test. It was noted that strains T7 and M2 showed a greater emulsification activity value (54.62 and 56.01%, respectively). Higher emulsification activity, foaming, and drop-collapse test value indicated that strains T7 and M2 could be promising isolates for biosurfactant production.

### Biodegradation of chlorpyrifos in different media

**FIGURE 3:**
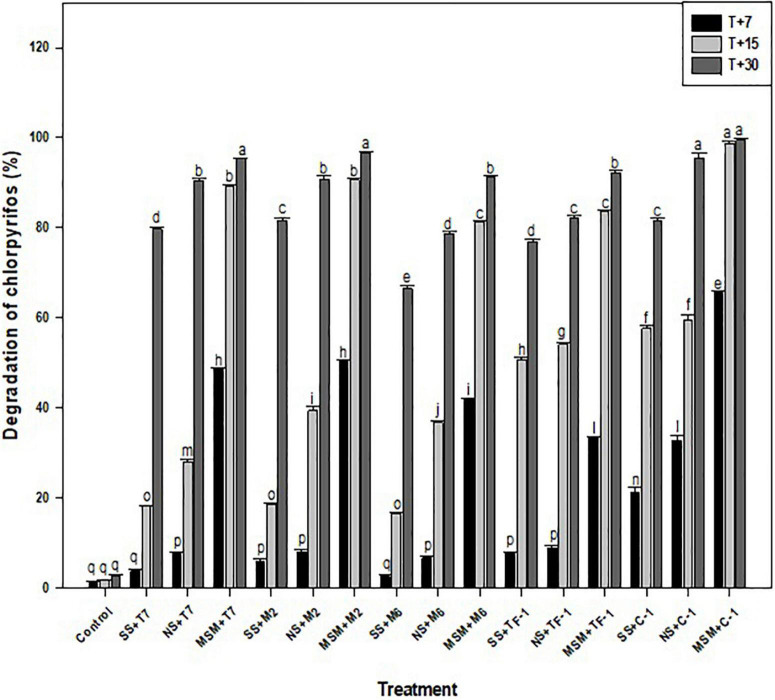
Chlorpyrifos biodegradation in different media at different time intervals. Data were the average of five replicates (*n* = 5). Bars represent standard error. Significant differences based on the analysis variance (ANOVA) are shown by different letters above the error bars, followed by the *post-hoc* DMRT test (*p* ≤ 0.05) using the software SPSS.

Chlorpyrifos biodegradation was performed with developed consortium ERM C-1 and with individual strains in different media (i.e., mineral salt medium, natural soil, and sterile soil) at different time (7, 15, and 30 days) intervals. The results revealed that in the control treatment, 100% chlorpyrifos was available in the MS medium as well as in natural and sterile soil systems at zero days of incubation. However, over the time period, abiotic degradation of chlorpyrifos was observed and recorded as 2.07 ± 0.01, 3.57 ± 0.02, and 5.32 ± 0.02% at 7, 15, and 30 days, respectively. The abiotic degradation of chlorpyrifos was taken into consideration to calculate the overall percentage of chlorpyrifos biodegradation at different treatments. Results showed the decrease in chlorpyrifos concentration (500 mg L^–1^) was started on the 7th day of incubation, followed by full disappearance on the 30th day of incubation in liquid MS medium with ERM C-1 consortium treatment (Figure 3). Results from individual strains revealed that strains T7, M2, M6, and TF1 degraded 95.39 ± 1.05, 96.69 ± 1.21, 91.77 ± 1.11, and 92.97 ± 1.04% of chlorpyrifos, respectively, in liquid MS medium after 30 days of incubation. Furthermore, in different soil systems, 98.58 ± 1.11 and 92.16 ± 0.18% degradation values were noted in natural and sterile soil systems with consortium ERM C-1 treatment, respectively, after 30 days of incubation. The biodegradation patterns of individual strains in natural and sterile soil systems revealed that strain T7 degraded 79.51 ± 0.12 and 90.15 ± 0.27% of chlorpyrifos in sterile and natural soils, respectively, after 30 days of incubation. Strain M2 degraded 82.55 ± 0.22 and 91.89 ± 0.25% of chlorpyrifos in sterile and natural soil systems after 30 days of incubation. In the case of strains M6 and TF1, 65.31 ± 0.11 and 77.72 ± 0.16% values of chlorpyrifos degradation were recorded in the sterile soil system after 30 days of incubation, respectively. In the natural soil system, strains M6 and TF1 degraded 79.25 ± 0.13 and 83.15 ± 0.19%, respectively, at 30 days of incubation (Figure 3). The observed results clearly highlighted the ability of all the four strains to utilize chlorpyrifos as sole carbon and phosphorous source for their growth and metabolism. Moreover, GC-MS analysis of chlorpyrifos biodegradation in MS medium, sterile soil, and natural soil detected different intermediate compounds during the biodegradation process. The GC-MS analysis also revealed there was no TCP formation during the biodegradation of chlorpyrifos in any treatment after 30 days of incubation.

### Biodegradation kinetics of chlorpyrifos in different media

The degradation kinetics of chlorpyrifos was assessed in three different media: MS medium, sterile soil, and natural soil systems. The degradation constant (*k* day^–1^) and half-life (*t*_1/2_) of chlorpyrifos in different media with different treatments (T7, M2, M6, TF1, and ERM C-1) were determined with the help of first-order biodegradation kinetics and linear model. The recorded results are depicted in Table 2. The theoretical half-life (*t*_1/2_) of chlorpyrifos in the MS medium with control treatment was 229 days, while it was calculated as 3.5 days with consortium ERM C-1 in the MS medium. Furthermore, in natural and sterile soil systems, the half-life of chlorpyrifos was determined at 121 and 139 days with control treatment, respectively. However, soil treated with ERM C-1 recorded a half-life of 12 and 17 days in natural and sterile soil systems, respectively.

**TABLE 2:** Chlorpyrifos degradation kinetics and half-life (*t*_1/2_) in natural (NSS), sterile soil (SS), and mineral salt medium (MSM).

Treatments	Regression equation	k^(day–1)^	R^2^	*t*_1/2_ (days)
SS + CHL	ln(Ct/C_0_) = −0.011x + 4.981	0.008 ± 0.003*^a^*	0.935 ± 0.003*^a^*	139.233 ± 0.33*^f^*
NSS + CHL	ln(Ct/C_0_) = −0.012x + 4.977	0.007 ± 0.002*^a^*	0.929 ± 0.002*^a^*	121.447 ± 0.31*^f^*
MSM + CHL	ln(Ct/C_0_) = −0.0022x + 4.998	0.002 ± 0.001*^a^*	0.910 ± 0.003*^a^*	229.753 ± 0.45*^g^*
SS + CHL + T7	ln(Ct/C_0_) = −0.019x + 4.968	0.012 ± 0.002*^c^*	0.980 ± 0.001*^c^*	18.131 ± 0.18*^d^*
NSS + CHL + T7	ln(Ct/C_0_) = −0.018x + 4.955	0.013 ± 0.002*^c^*	0.981 ± 0.002*^c^*	14.652 ± 0.15*^c^*
MSM + CHL + T7	ln(Ct/C_0_) = −0.019x + 4.858	0.014 ± 0.001*^d^*	0.999 ± 0.001*^d^*	5.517 ± 0.04*^a^*
SS + CHL + M2	ln(Ct/C_0_) = −0.039x + 4.968	0.013 ± 0.001*^c^*	0.961 ± 0.002*^b^*	16.611 ± 0.12*^c^*
NSS + CHL + M2	ln(Ct/C_0_) = −0.038x + 4.968	0.012 ± 0.002*^c^*	0.975 ± 0.006*^b^*	13.174 ± 0.11*^c^*
MSM + CHL + M2	ln(Ct/C_0_) = −0.039x + 4.858	0.014 ± 0.002*^d^*	0.985 ± 0.006*^c^*	5.958 ± 0.03*^b^*
SS + CHL + M6	ln(Ct/C_0_) = −0.013x + 4.945	0.011 ± 0.003*^b^*	0.991 ± 0.002*^d^*	19.131 ± 0.18*^d^*
NSS + CHL + M6	ln(Ct/C_0_) = −0.012x + 4.829	0.012 ± 0.003*^c^*	0.978 ± 0.003*^b^*	14.865 ± 0.12*^c^*
MSM + CHL + M6	ln(Ct/C_0_) = −0.011x + 4.747	0.012 ± 0.003*^c^*	0.991 ± 0.001*^d^*	06.517 ± 0.04*^b^*
SS + CHL + TF1	ln(Ct/C_0_) = −0.049x + 4.878	0.010 ± 0.004*^b^*	0.970 ± 0.004*^b^*	23.522 ± 0.21*^e^*
NSS + CHL + TF1	ln(Ct/C_0_) = −0.039x + 4.858	0.010 ± 0.003*^b^*	0.931 ± 0.005*^a^*	21.747 ± 0.19*^e^*
MSM + CHL + TF1	ln(Ct/C_0_) = −0.051x + 4.823	0.011 ± 0.003*^b^*	0.971 ± 0.005*^b^*	06.147 ± 0.07*^b^*
SS + CHL + ERM C-1	ln(Ct/C_0_) = −0.017x + 4.868	0.016 ± 0.001*^e^*	0.991 ± 0.001*^d^*	17.678 ± 0.13*^d^*
NSS + CHL + ERM C-1	ln(Ct/C_0_) = −0.018x + 4.822	0.018 ± 0.001*^e^*	0.984 ± 0.004*^c^*	12.746 ± 0.10*^c^*
MSM + CHL + ERM C-1	ln(Ct/C_0_) = −0.016x + 4.629	0.019 ± 0.001*^e^*	0.997 ± 0.001*^d^*	03.513 ± 0.02*^a^*

^a,b,c,d,e,f,g^Indicates significant mean difference between the groups or variables.

### Soil enzyme dynamics of chlorpyrifos-treated soils

**FIGURE 4:**
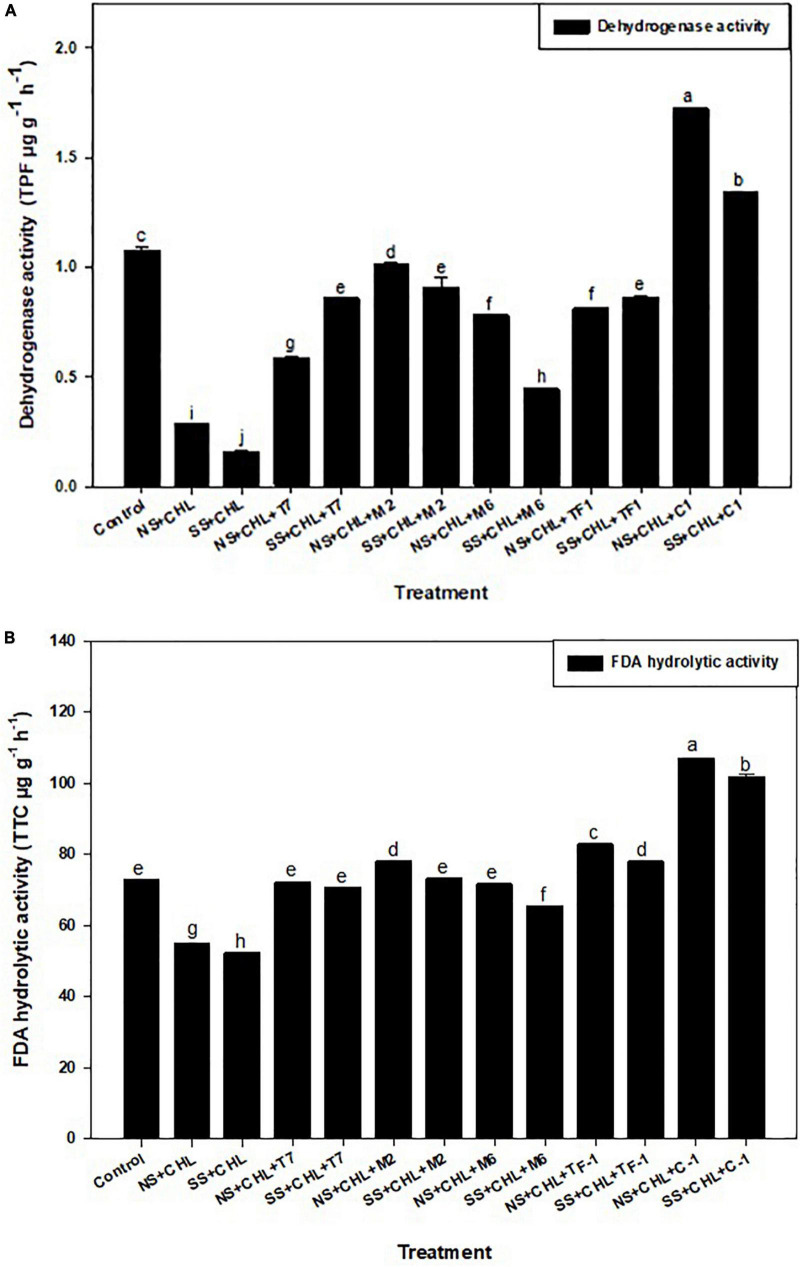
**(A)** Dehydrogenase activity of the soil treated by different strains and developed consortium in sterile and natural conditions. The recorded data were the average of five replicates (*n* = 5). Bars represent the standard error. Significant differences based on the analysis variance (ANOVA) are shown by different letters above the error bars, followed by the *post-hoc* DMRT test (*p* ≤ 0.05) using the software SPSS. **(B)** Fluorescein diacetate (FDA) hydrolysis activity of the soil treated with different strains and developed consortium in sterile and natural conditions. The recorded data were the average of five replicates (*n* = 5). Bars represent standard error. Significant differences based on the analysis variance (ANOVA) are shown by different letters above the error bars, followed by the *post-hoc* DMRT test (*p* ≤ 0.05) using the software SPSS.

Two soil enzymes (DHA and FDA) were estimated in chlorpyrifos contaminated soil. Results revealed that chlorpyrifos decreased the soil DHA and FDA activity when compared to the control (natural) soil. Furthermore, it was observed that when chlorpyrifos contaminated soil was treated with different microbial strains (T7, M2, M6, and TF1) and developed consortium (ERM C-1), the activity of DHA and FDA was significantly (≤0.05) increased (Figures 4A,B). The increase in DHA activity when compared to their respective controls was 52.07 ± 0.12 and 27.66 ± 0.11% when treated with consortium ERM C-1 in natural and sterile soils, respectively. In the case of FDA, 72.09 ± 0.27 and 71.34 ± 0.25% increase in the activity was observed in natural and sterile soils, respectively, with ERM C-1 treatment when compared to the corresponding control. The observed results clearly indicated that chlorpyrifos-degrading microbial consortium ERM C-1 significantly increased the soil enzyme activity.

### Heat map analysis of different biodegradation patterns of chlorpyrifos contaminated soil

**FIGURE 5:**
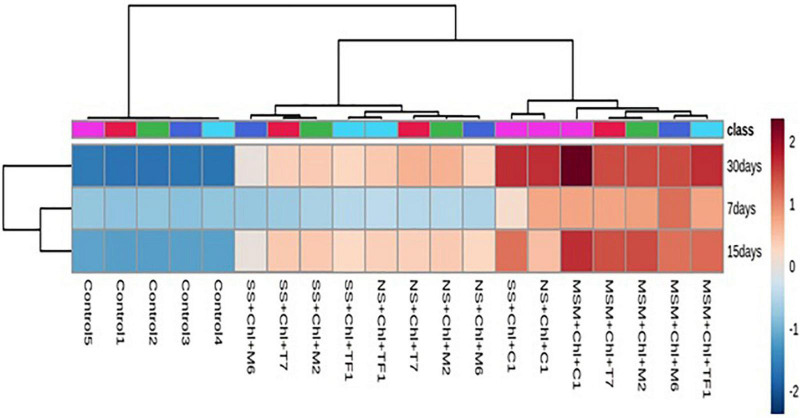
Heat map analysis of chlorpyrifos biodegradation by individual strains and developed consortium (ERM C-1) at different time intervals.

To analyze the degradation pattern in different media with different treatments, a heat map with clustal correlation analysis was performed. Results revealed that five different groups (ERM C-1, T7, M2, M6, and TF1) were constructed representing each treatment with different media (MSM, NS, and SS) at different incubation periods (7, 15, and 30 days). It was observed that when chlorpyrifos was treated with different strains and ERM C-1 in the MS medium and incubated for 30 days, the degradation pattern was ERM C-1>TF1>T7>M2>M6. Furthermore, the complete disappearance of chlorpyrifos with ERM C-1 treatment was observed in the MS medium after 30 days of incubation (Figure 5), while in the case of natural soil, the chlorpyrifos degradation pattern was ERM C-1>T7>M2>TF1>M6 after 30 days of incubation. In sterile soil, chlorpyrifos degradation pattern was observed as ERM C-1>M2>T7>TF1>M6 after 30 days of incubation. Consortium ERM C-1 was found to be most effective in chlorpyrifos degradation in both natural and sterile soil treatments after 30 days of incubation.

## Discussion

Chlorpyrifos is an organophosphate insecticide that is commonly used to control pests in vegetable and fruit crops (Raj et al., 2021). The increased use of this pesticide poses a serious impact on the ecosystem. As a result, reclamation of highly polluted arable land is urgently required (Bose et al., 2021). Physical and chemical procedures are being employed to detoxify numerous locations across the world, but they are costly and contaminate the same areas with secondary contaminants (Huang et al., 2021). In contrast, microbiological techniques are inexpensive, environmentally benign, and economically sound (Uniyal et al., 2021). Earlier studies of microbial chlorpyrifos remediation revealed that rhizosphere microbes are the most willing bio-agents because they produce extracellular enzymes that can participate in the bioremediation of various recalcitrant/xenobiotic compounds and support bacterial and fungal survival by providing essential nutrients (Bose et al., 2021; Raj et al., 2021; Uniyal et al., 2021). In this study, 21 chlorpyrifos utilizing/resistant bacteria and four fungal isolates were recovered from the agricultural soils contaminated by different pesticides. Furthermore, three bacterial and one fungal strain were thriving at 500 mg L^–1^ of chlorpyrifos concentration and showed great plant growth promotion and laccase activity in the presence of chlorpyrifos. Recently, Kumar et al. (2021c) reported the use of *Klebsiella pneumoniae* M11 for chlorpyrifos biodegradation and plant growth promotion. Ambreen and Yasmin (2021) isolated *Bacillus thuringiensis* MB497 from the agricultural soil which showed resistance to 200 mg L^–1^ concentration of chlorpyrifos and degraded up to 99% of chlorpyrifos in the M9 medium. Similarly, Yadav et al. (2020) reported that *Alcaligenes faecalis* (NBRI OSS2-5) degraded 94% of chlorpyrifos in MS medium at 10 days of incubation and produced exopolysaccharides which have good plant growth-promoting characteristics. Apart from bacteria, fungal strains have also been well-reported for chlorpyrifos degradation and laccase production. Recently, Kumar et al. (2021) reported that two fungal strains (*B. spectabilis* C1 and *A. fumigatus* C3) and their consortium degraded chlorpyrifos up to 96.7, 93.45, and 98.4%, respectively, in Czapek Dox medium (CDM) at 30 days of incubation, and individual strains were also able to produce laccase (2.23 and 1.74 U mL^–1^, respectively). In this study, three bacterial strains, *P. putida* (T7), *Pseudomonas aeruginosa* (M2), and *Klebsiella pneumoniae* (M6), and one fungal strain, *A. terreus* (TF1), showed the potential to degrade chlorpyrifos in MS medium, natural soil, and sterile soil up to 95–100% and showed great plant growth-promoting traits, which are noteworthy in this regard. Moreover, when the aforementioned strains were mixed together and developed as a consortium, the metabolic burden of the single strain splits into several parts and showed best results than the individual strains alone. Recently, Uniyal et al. (2021) have developed a consortium ECO-M comprising different bacterial strains (*Agrobacterium tumefaciens* ECO1, *Cellulosimicrobium funkei* ECO2, *Shinella zoogloeoides* ECO3, and *Bacillus aryabhattai* ECO4) for chlorpyrifos degradation in low-temperature mountainous agriculture system and reported 21.6–94.3% of degradation in 30 days of incubation. Similarly, in the present study, two different domain microorganisms (bacteria and fungus) were mixed together and developed as consortium ERM C-1 for chlorpyrifos biodegradation and plant growth promotion to reduce pesticide load and enhance crop production in subtropical agricultural lands.

The chlorpyrifos biodegradation was examined at 7-, 15-, and 30-day intervals in different media, such as minimal salt, natural soil, and sterile soil. The maximum chlorpyrifos degradation was recorded in the group treated with consortium rather than with individual strains, which might be due to the co-metabolism of strains where each strain produced a combination of catabolic enzymes responsible for more rapid and enhanced biodegradation of chlorpyrifos. Moreover, it might be attributed to the rapid growth of microbial strains during the first 15 days of the incubation period, or to the fact that a multi-strain consortium has a more efficient enzymatic system for chlorpyrifos biodegradation than the individual strain. The induction or activation of certain genes responsible for the production of catabolic enzymes in consortium treatment probably hydrolyzed the P–O ester bond causing rapid biodegradation in a short period of time, which might be another reason for chlorpyrifos biodegradation (Sun et al., 2020). The obtained results were encouraging and showed complete degradation in MS medium at 30 days of incubation while 98.56 ± 2.1 and 96 ± 1.7% in natural and sterile soils, respectively, at 30 days of incubation, which is more relevant than the previous studies. Similarly, Elshikh et al. (2021) formulated a bacterial consortium using two bacterial strains (*B. cereus* CP6 and *K. pneumoniae* CP19) and reported 93.4 ± 2.8% chlorpyrifos degradation by consortium treatment in liquid culture while 94.5 ± 3.3% in the soil at 16 days of the time interval.

Plant growth-promoting characteristics are the special feature found in plant rhizosphere micro-organisms and bulk agricultural soil, and some of these microorganisms also have pesticide-degrading abilities as a consequence of constant exposure to these substances (Kumar et al., 2021a,b). Previously, different microorganisms responsible for chlorpyrifos breakdown were identified from soil enrichment culture (Zhao et al., 2014; Govarthanan et al., 2020). In this study, all four strains (T7, M2, M6, and TF1) showed the best PGP traits, such as IAA, HCN, and NH_4_ production and phosphate, potassium, and zinc solubilization ability in lab conditions. In the same way, Govarthanan et al. (2020) isolated a chlorpyrifos-degrading potent plant growth-promoting psychrophilic bacteria *Shewanella* sp. BT05 from brackish water, which showed IAA, HCN, and siderophore production and phosphate solubilization characteristics. Similarly, Zhao et al. (2014) reported *Acinetobacter calcoaceticus* D10 had the ability to degrade chlorpyrifos and showed IAA, siderophore, and phosphate solubilization activity in lab conditions.

Enzyme tests are used to investigate the mechanism by which microbes degrade complex substances. A polyphenol oxidase enzyme, laccase, has previously been reported to attack complex aromatic chemicals and produce simpler compounds, and some microbes have already been reported to be laccase producers and their linkage to chlorpyrifos biodegradation (Liu et al., 2016; Das et al., 2020; Srinivasan et al., 2020; Kumar et al., 2021). In this connection, different researchers have conducted studies on fungal and bacterial species capable of producing laccase and reported that *Bacillus halodurans*, *Azospirillum lipoferum*, *Pseudomonas desmolyticum*, *Bacillus pumilus*, *Bacillus subtilis*, *P. putida, B. spectabilis*, and *A. fumigatus* strains produced laccase enzyme (Gangola et al., 2018; Kumar et al., 2021). In this investigation, higher laccase production by consortium ERM C-1 than by individual strain might possibly be the reason for a greater chlorpyrifos degradation ability of ERM C-1. Srinivasan et al. (2020) reported *Bacillus* sp. for laccase production and chlorpyrifos degradation. Similarly, an engineered *P. putida* MB285 has been reported by Liu et al. (2016) for laccase production and complete chlorpyrifos degradation.

Biosurfactant is an amphipathic molecule made up of hydrophobic and hydrophilic moieties (Lal et al., 2018; Singh et al., 2018). *Bacillus* and *Pseudomonas* have previously been identified as potential biosurfactant producers for the removal of hazardous organic compounds and heavy metals from contaminated environments (Ambust et al., 2021; Ratna and Kumar, 2022). In this study, strains T7 and M2 showed biosurfactant production, and these strains were combined with other strains and included in consortium ERM C-1.

Pesticide biodegradation in the soil is an enzyme-driven transformation of organic compounds in a certain time duration. First-order degradation kinetics is frequently used to mimic the decline of residual pesticide mass in the soil system after its application (Bollag and Liu, 1990). The residual mass of pesticide diminishes exponentially with time “t” and the rate of degradation “*k”* or half-life continuously changes with the degradation process (Dykaar and Kitanidis, 1996). In this study, the values of degradation constant *k* were low and half-life (*t*_1/2_) values were higher in the control condition, while an increase in *k* and decrease in *t*_1/2_ values were observed with ERM C-1 treatment, indicating the rapid chlorpyrifos degradation in different media, which might be due to enzyme-catalyzed transformation of chlorpyrifos. The *k* and *t*_1/2_ values of individual strains were lower than ERM C-1, indicating the combined effects of different enzymes present in the consortium treatment. Recently, Gaonkar et al. (2019) studied the biodegradation kinetics of dichlorvos and chlorpyrifos with *Pseudomonas aeruginosa* and *Taonella mepensis* in liquid MS medium, and similar findings were reported. Moreover, Kumar et al. (2021) performed biodegradation kinetics of chlorpyrifos with two fungal strains and reported decreased values of *k* and *t*_1/2_ with consortium treatments.

Soil dehydrogenase (DHA) and fluorescein diacetate (FDA) activities are directly related to soil microbial activities and are considered as soil microbial indicators. DHA activity exists in soil as part of the oxidative processes occurring within living cells, and its measurement reflects intracellular enzymes solely; therefore, it is considered a direct indicator of microbial activity. However, FDA activity is a non-specific assay in which lipase, esterase, and protease classes of enzymes hydrolyze fluorescein by cleavage of the ring, so it is assumed that they may also take part in the cleavage of P-O bonds present in chlorpyrifos ring. In this study, an increase in DHA and FDA activities with ERM C-1 treatment indicated that microbial consortium significantly increased the soil enzyme activity. Similar findings were reported by Kumari et al. (2021) with fipronil and atrazine degradation using biochip mixed biomixtures. Medo et al. (2021) reported reduced DHA and FDA activity with the application of two insecticides dimethachlor and linuron.

### Proposed working mechanisms of ERM C-1

**FIGURE 6:**
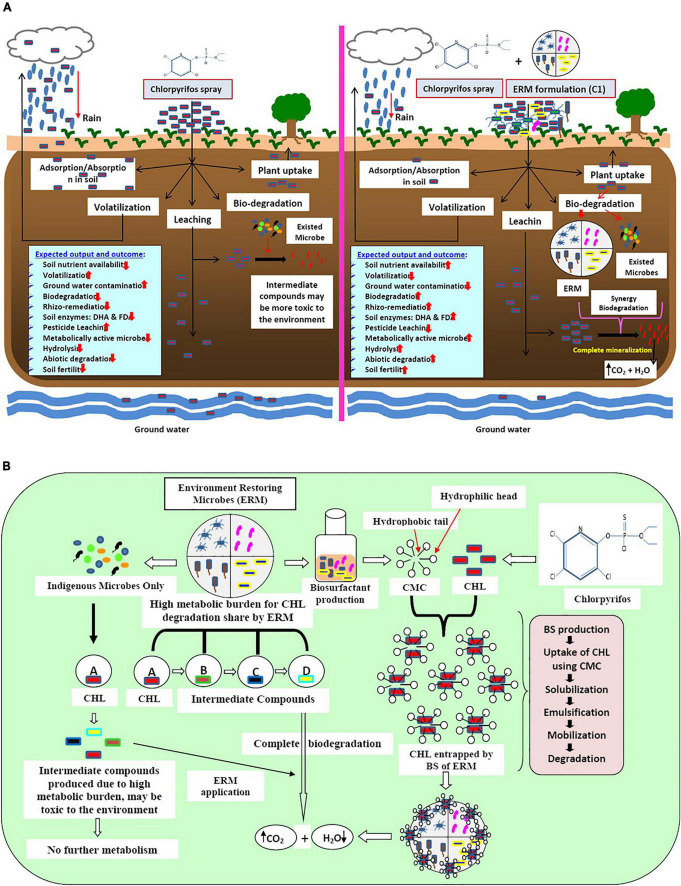
**(A)** Schematic diagram of the impact of pesticide (chlorpyrifos) and microbes (ERM- environment restoring microbes-C-1 formulation and already exited microbes) on soil, plant, and environment. **(B)** Mechanisms of action of bio-surfactant producing environment restoring microbes (ERM) in chlorpyrifos (CHL) biodegradation.

The application of chlorpyrifos in the field leads to various negative impacts on soil, water, and plant systems. Due to high hydrophobicity, chlorpyrifos adheres to the soil particles and forms clumps that entrap nutrients and restrict its movement toward the plant root. However, some amount of chlorpyrifos evaporates with rainwater while some quantity percolates down and contaminates groundwater. Consequently, the bioavailability of chlorpyrifos reduces greatly. In addition to all the negative impacts of chlorpyrifos on the whole system after its application, a compromised soil ecosystem has been generated in terms of plant nutrient uptake, leaching, and atmospheric pollution. However, trace quantities of chlorpyrifos remain available for microbial action leading to the partial degradation by indigenous microflora, subsequently resulting in the production of an intermediate compound like 3,5,6-trichloro pyridine-2-phenol (TCP), which showed a more toxic effect on the ecosystem than its parent compound. Production of such intermediate compounds is due to the reduction of metabolically active indigenous microbes (less metabolic versatility). On the other hand, when microbial formulation ERM C-1 was used, the produced intermediate compound was utilized immediately by high metabolically active microbes and metabolized completely into carbon dioxide and water. Simultaneously, ERM C-1 reduced the residual effect of chlorpyrifos and can establish better soil and plant health with high soil nutrient availability and soil enzymatic activities (Figure 6A).

### Functioning of ERM C-1

ERM C-1 is constituted by high metabolically active microbes toward chlorpyrifos biodegradation, and hence enhances the bioavailability of chlorpyrifos by producing a special compound called biosurfactants (BS). Due to the high hydrophobic nature of chlorpyrifos, it persists in nature for a long duration. BS produced by ERM C-1 showed amphipathic nature and had a hydrophobic head and a hydrophilic tail. The hydrophobic part interacts with the benzene ring of chlorpyrifos, making it available for microbial attack by encapsulation process via critical micelle formation. When chlorpyrifos is available for ERM C-1 activity, the production of ring-opening enzymes like deoxygenase and other hydrolytic enzymes (hydrolases, oxidases, etc.) is initiated, and after the mineralization process, chlorpyrifos gets converted into CO_2_ and H_2_O. On the other hand, already existing but metabolically less versatile microbes degraded chlorpyrifos partially and produced intermediate compounds like TCP, which is more toxic than its parent compound. Excessive chlorpyrifos application caused a high metabolic burden to already existing microbes in the system which degrade chlorpyrifos partially, and toxic intermediate compounds prevail in the system. To address this problem, ERM C-1 application can be the best option because it showed the co-metabolism system by sharing metabolic burden through different microbes (Figure 6B). Therefore, the ERM C-1 application is recommended by this study for efficiently decreasing the chlorpyrifos concentration in the soil environment that has been affected by the excessive application of chlorpyrifos.

## Conclusion

Prior research has not paid much attention to the question of whether combining the two domains of microorganisms (bacteria and fungus) is important to increase metabolic versatility for contaminant/pollutant removal. This study will look at novel ideas linked to the interactions between two domains of microbes in order to identify suitable co-metabolism methods for removing chlorpyrifos from the environment. The microbial strains (T7, M2, M6, and TF1) isolated from agriculture fields were able to thrive in chlorpyrifos (500 mg L^–1^)-supplemented medium. All four strains were used to develop consortium ERM C-1, which successfully degraded 100% of chlorpyrifos in the liquid MS medium. All the strains were able to produce extracellular laccase enzyme, which aided chlorpyrifos biodegradation. Plant growth promotion activities of the strains showed great potential, which can be an extra benefit to chlorpyrifos contaminated soil that is used for crop production. As shown by the kinetic constants, ERM C-1 demonstrated distinct capacities for chlorpyrifos degradation in different media. An *in-situ* pot experiment with natural and sterile soils, spiked with 500 mg kg^–1^ chlorpyrifos, revealed 98.58 ± 1.11 and 92.16 ± 0.18% degradation with ERM C-1 consortium. The conversion of chlorpyrifos into various metabolites was discovered using HPLC and GC-MS analyses. This is the first study to investigate chlorpyrifos biodegradation by employing a mixed fungal and bacterial consortia with a strong biodegradation capacity. In this study, *A. terreus* TF1 emerged as a powerful fungus that revealed great results and could be employed in a variety of bioremediation experiments. The consortium ERM C-1 can be further applied to degrade harmful compounds from a variety of sources. Further research can be conducted in the future by employing ERM C-1 on a broad scale with varied pesticide concentrations and crops in different regions. This research might aid in the practical application of consortium ERM C-1 in the removal of chlorpyrifos in polluted environments.

## Data availability statement

The datasets presented in this study can be found in online repositories. The names of the repository/repositories and accession number(s) can be found at: https://www.ncbi.nlm.nih.gov/genbank/ (MW172266, MW228061, and MZ 268151).

## Author contributions

GK: conceptualization, methodology, writing, and analysis. SL: writing, editing, methodology, and analysis. SS: analysis of results. SM, PS, PC, and AB: methodology and editing. NG: conceptualization and methodology. All authors contributed to the article and approved the submitted version.

## Footnotes

1
www.ncbi.nlm.nih.gov
