# Supplementary material for: Mechanism and kinetics of chlorpyrifos co-metabolism by using environment restoring microbes isolated from rhizosphere of horticultural crops under subtropics
Source: Front Microbiol. 2022 Jul 26;13:891870. doi: 10.3389/fmicb.2022.891870 (PMC9360973; doi:10.3389/fmicb.2022.891870)
Supplement: Supplementary file 1 [file Data_Sheet_1.docx]

*Pseudomonas mosselii* str. IRQBAS129 (LC648433)

Uncultured *Pseudomonas* *sp.* clone B-LO-T1_OTU11 (FM204971)

*Pseudomonas plecoglossicida* str. RTE-E1 (LC572260)

*Pseudomonas sp.* str. Marseille-Q3773 (OU564159)

*Pseudomonas fluorescens* str. TG5-2 (LC575126)

*Pseudomonas putida* str. JCM 20228 (LC654885)

*Pseudomonas putida* str. RL-JY1 (MK101056)

***Pseudomonas putida* str. T7 (MW172266)**

*Pseudomonas putida* str. RS-17 (DQ112329)

*Pseudomonas putida* str. SF84B (LC567891)

*Pseudomonas putida* str. CT3 (MH553084)

*Pseudomonas moraviensis* isolate BE8 (LR214463)

*Pseudomonas reactans* str. NO8 (FJ972537)

*Pseudomonas azotoformans* str. JCM 20222 (LC654882)

*Pseudomonas lactis* isolate MTR3A (LR214465)

*Pseudomonas cyclaminis* str. MAFF 301453 (LC582670)

*Pseudomonas marginalis* str. ATCC 10844 (AB021401)

*Pseudomonas lundensis* str. ATCC 49968 (AB021395)

*Pseudomonas savastanoi* str. ATCC 13522 (AB021402)

*Pseudomonas meliae* str. MAFF 301463 (AB021382)

*Pseudomonas ficuserectae* str. JCM 2400 (AB021378)

*Pseudomonas xanthomarina* isolate 0911TES21U2 (LN774354)

*Pseudomonas zhaodongensis* isolate Hvs53 (OU707438)

*Pseudomonas stutzeri* str. JCM 20400 (LC654903)

*Pseudomonas oleovorans* str. JCM 13978 (LC508004)

*Pseudomonas alcaliphila* str. CI12 (HG796164)

*Pseudomonas pseudoalcaligenes* str. JCM 5968 (AB021379)

*Pseudomonas anguilliseptica* str. NCIMB 1949 (AB021376)

*Pseudomonas tohonis* str. TUM18999 (LC645211)

*Pseudomonas nitroreducens* str. TG10-1 (LC575130)

*Pseudomonas citronellolis* str. ATCC 13674 (AB021396)

*Pseudomonas aeruginosa* str. JCM 20301 (LC654886)

*Pseudomonas aeruginosa* str. IRQBAS137 (LC648441)

*Pseudomonas aeruginosa* str. K2 (FJ972528)

*Pseudomonas aeruginosa* str. NO6 (FJ972534)

*Pseudomonas aeruginosa* str. CJM (FJ972527)

*Pseudomonas aeruginosa* str. TB11 (LC597461)

*Pseudomonas aeruginosa* str. 5PD4 (OK668300)

***Pseudomonas aeruginosa* str. M2 (MW228078**)

*E.coli* ATCC 11775T (X80725)

86

99

99

79

78

97

99

47

41

84

98

82

48

83

29

18

66

98

78

70

90

68

0.02

**Fig. S1.** Phylogenetic tree constructed from the 16S ribosomal RNA of strains T7, and M2 and related organisms using Maximum Likelihood algorithm from an alignment of 1284 nucleotides. Accession numbers of corresponding sequences are given in parentheses, and scale bar represents 1 base substitution per 20 nucleotide positions. The bootstrap probabilities calculated from 1,000 replications. E. coli ATCC 11775T was taken as an out-group
