# Supplementary material for: Mechanism and kinetics of chlorpyrifos co-metabolism by using environment restoring microbes isolated from rhizosphere of horticultural crops under subtropics
Source: Front Microbiol. 2022 Jul 26;13:891870. doi: 10.3389/fmicb.2022.891870 (PMC9360973; doi:10.3389/fmicb.2022.891870)
Supplement: Supplementary file 2 [file Data_Sheet_2.docx]

***Klebsiella pneumoniae* str. M6 (MW228061)**

*Klebsiella pneumoniae* str. KPWIQ25 (MT102629)

*Klebsiella pneumoniae* str. HF4-SOB (LC617176)

*Klebsiella milletis* (AY217656)

*Klebsiella granulomatis* str. gall248 (MW435512)

*Klebsiella sp.* str. IPF4-SOB (LC617179)

*Klebsiella quasivariicola* str. AKP B2 (MW221385)

*Klebsiella sp.* str. UKT3 (LC636411)

*Klebsiella senegalensis* (AY217655)

*Klebsiella steroids* str. NCIM 2957 (KR078394)

*Klebsiella quasipneumoniae* str. UKT75 (LC636413)

*Klebsiella variicola* str. PCH427 (MZ914413)

*Klebsiella grimontii* str. WA12 (MZ430132)

Uncultured *Klebsiella sp.* (AB188790)

*Klebsiella michiganensis* str. Mw1 (LC191534)

*Klebsiella pasteurii* str. Ko4 (MN104672)

*Klebsiella spallanzanii* str. SB6411_SPARK775C1T_Ko3 (MN091365)

*Klebsiella oxytoca* isolate HL1HP15 (LT221131)

Uncultured *Klebsiella sp.* clone W1SH16 (LT576226)

Uncultured *Klebsiella sp.* clone W4R26 (LT576221)

*Klebsiella aerogenes* str. CUMB TP-07 (OK605774)

Uncultured *Klebsiella sp.* clone JUR2 (MT981257)

*Klebsiella huaxiensis* str. Ko8 (MN104673)

*Bacillus subtilis* subsp. *spizizenii* (AF074970)

100

60

38

73

37

32

19

44

62

58

65

16

0.05

**Fig. S2.** Phylogenetic tree constructed from the 16S ribosomal RNA of strains M6 and related organisms using Maximum Likelihood algorithm from an alignment of 933 nucleotides. Accession numbers of corresponding sequences are given in parentheses, and scale bar represents 1 base substitution per 50 nucleotide positions. The bootstrap probabilities calculated from 1,000 replications. *Bacillus subtilis* subsp. *spizizenii* was taken as an out-group
