# Supplementary material for: Mechanism and kinetics of chlorpyrifos co-metabolism by using environment restoring microbes isolated from rhizosphere of horticultural crops under subtropics
Source: Front Microbiol. 2022 Jul 26;13:891870. doi: 10.3389/fmicb.2022.891870 (PMC9360973; doi:10.3389/fmicb.2022.891870)
Supplement: Supplementary file 3 [file Data_Sheet_3.docx]

**
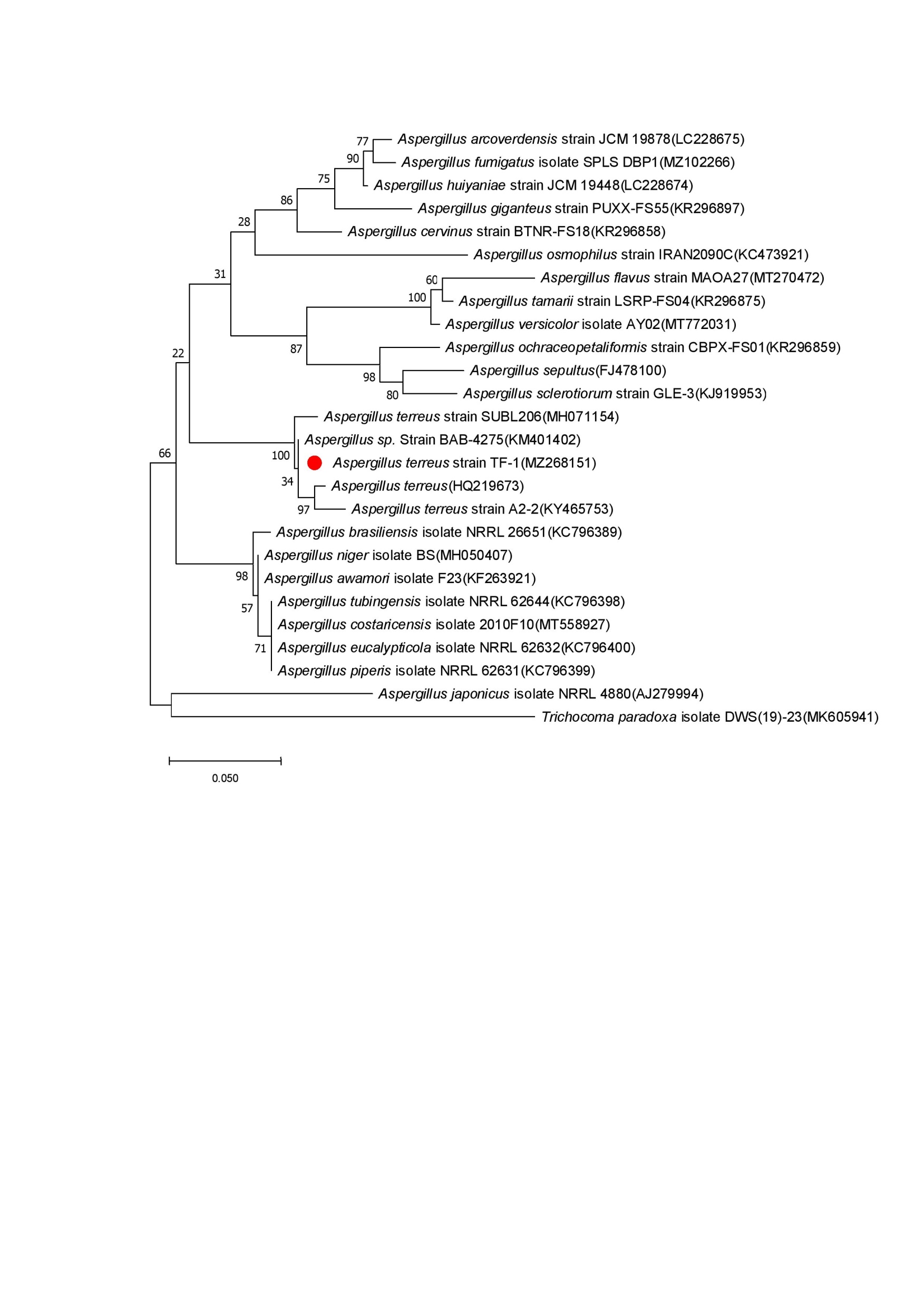
**

**Fig. S3.** Phylogenetic tree constructed from the internal transcribe spacer 1 of 5.8S ribosomal RNA of strains TF-1 and related organisms constructed using Maximum Likelihood algorithm from an alignment of 551 nucleotides. Accession numbers of corresponding sequences are given in parentheses, and scale bar represents 1 base substitution per 50 nucleotide positions. The bootstrap probabilities calculated from 1,000 replications. *Trichocoma paradoxa* isolate DWS (19)-23 was taken as an out-group.
